# Integration and Analysis of Omics Data Using Genome-Scale Metabolic Models

**DOI:** 10.3390/metabo14110595

**Published:** 2024-11-05

**Authors:** Miha Moškon, Tadeja Režen

**Affiliations:** 1 Faculty of Computer and Information Science, University of Ljubljana, 1000 Ljubljana, Slovenia; 2 Centre for Functional Genomics and Bio-Chips, Institute for Biochemistry and Molecular Genetics, Faculty of Medicine, University of Ljubljana, 1000 Ljubljana, Slovenia; tadeja.rezen@mf.uni-lj.si

Constraint-based modelling and genome-scale metabolic models (GEMs) have been used extensively to analyze omics data, providing a mechanistic perspective on complex metabolic systems and networks [1]. Since they were first introduced in 1999, the number and complexity of GEMs have steadily increased [2]. This would not have been possible without close collaboration between experimentalists, who provided new techniques for the acquisition of high-throughput data, and a community of in silico researchers, who provided computational methods and algorithms that have also been incorporated into different computational toolboxes, such as COBRA [3], COBRApy [4], and RAVEN [5]. These toolboxes provide tools and algorithms not only for reconstructing models (e.g., using KEGG data [6]) but also for simulating, analysing, and adapting GEMs context-specifically using experimental high-throughput data [7].

This Special Issue of *Metabolites* is dedicated to original scientific and review papers that detail recent advances in the reconstruction, reproducibility, validation, and analysis of context-specific GEMs. The contributions included focus both on how GEMs have been applied within systems and personalised medicine and the methodological advances that have occurred in analysing metabolic pathways.

Understanding the effects of cold storage on human platelets is essential for their use in medicine and thus the main focus of contribution 1. Jóhannsson et al. applied constraint-based modelling of metabolomic data to investigate how the temperature and duration of their storage affect the metabolic state of human platelets (contribution 1), showing that their metabolic state changes with time and temperature but that this dependence is complex and does not follow an Arrhenius-type relationship. A cell-scale model of the platelets revealed that oxidative metabolism is more sensitive to lower temperatures than glycolysis, with glycolysis contributing a higher percentage of ATP at cold temperatures than at body temperature.

Meanwhile, Mattei et al. focused on automatically identifying metabolic pathways related to the production and consumption of specific metabolites (contribution 2). These authors introduced the MetPath algorithm, a valuable tool for performing metabolic-network-based statistical analyses of high-throughput data. MetPath is able to identify metabolic pathways specific to condition-related metabolite production and consumption. Equally, the tool proposed can be used to perform differential analyses of gene expression data based on these pathways under various conditions.

Srivastava and Vinod report applying GEMs to studying the metabolic reprogramming of cancer cells and identifying their metabolic subtypes in contribution 3, applying the Human Metabolic Reaction (HMR) database 2.0 in combination with transcriptomics data on endometrial cancer cells retrieved from TCGA. Using non-negative matrix factorisation-based clustering of the top 1000 genes based on the median absolute deviation score, they identified two metabolic subtypes of endometrial cancer tumours with different patient survival outcomes and showed that these two metabolic subtypes were correlated with histological and clinical features and genomic alterations.

In contribution 4, Sen and Orešič describe various applications of GEMs to advancing precision medicine, especially in combination with popular machine learning approaches. They address context-specific genome-scale metabolic reconstructions using multi-omics data; modelling the interactions between gut microbial communities and host metabolism; and the use of machine learning for genome-scale metabolic modelling. In addition, they discuss the challenges in and future perspectives on improving the reliability and reproducibility of GEM-based predictions in precision medicine.

Several algorithms for extracting context-specific genome-scale metabolic models (GEMs) using various types of high-throughput omics data have been proposed in the past. However, each algorithm has its own advantages and disadvantages. The selection of the best algorithm usually depends on criteria such as the type of data, the domain of the data, and the specific research questions. Moškon and Režen provide a thorough review of these algorithms, referred to as model extraction methods, and discuss their application to identifying the metabolic signatures of COVID-19 (contribution 5).

Despite rapid advancements in systems biology and systems medicine in recent years, we are still a long way from fully understanding the dynamics of human cells. However, the approaches described in this Special Issue are a significant step towards this goal.
